# Health Tract No. 328

**Published:** 1868-06

**Authors:** 


HEALTH TRACT No. 328.

### BURYING ALIVE.

The great Washington and others of eminence, suffered through life with a fear of being buried alive ; most distressing narrations have been given from time to time about dead bodies being found turned over in their coffins ; such things may have taken place, but it is a very rare occurrence ; it is known that in some parts of Europe the corpse is placed in such a position, for several days, that if it should revive, a bell rope touches the hand and the alarm could be given to the whole neighborhood ; in one hundred years it did not occur, that one of these bells had been rung a single time. Still great efforts have been made by scientific men to discover some rule by which death may be infallibly indicated, for many years the French government have held out a standing reward of a large amount of money to any one who would discover and communicate a satisfactory test, other than that of actual decomposition, indicated by the skin turning to be black and blue and green which is conclusive on the subject ; but in cold weather this may not take place in many weeks, and to "keep the body" so long, would be inconvenient, and objectionable on several accounts. A method has recently been given to the French government which will probably take the prize ; hold a lighted candle to any portion of the body, a blister will soon rise ; if on puncture it gives out a fluid substance, death has not taken place ; if it emits air only, it is perfectly certain that life has become entirely extinct, for which we offer but one reason among others, in case of actual death the blood is congealed in a sense, there is no moisture, simply a little air, this being rarified under the flame, raises up the skin ; if there is life, the flame causes an inflammation and nature, in her alarm, sends increased material there for repair, a kind of glairy fluid, and this being sent there in excess, causes the skin to rise ; inability to feel the pulse or heart beat ; cold skin, no dew on a bit of glass, none of these are conclusive, as there has been life, when none of these were observed.

